# Tip60 complex binds to active Pol II promoters and a subset of enhancers and co-regulates the c-Myc network in mouse embryonic stem cells

**DOI:** 10.1186/s13072-015-0039-z

**Published:** 2015-11-06

**Authors:** Sarina Ravens, Changwei Yu, Tao Ye, Matthieu Stierle, Laszlo Tora

**Affiliations:** Cellular Signalling and Nuclear Dynamics Programme, Institut de Génétique et de Biologie Moléculaire et Cellulaire (IGBMC), CNRS UMR 7104, INSERM U964, Université de Strasbourg (UdS), BP 10142, 1 Rue Laurent Fries, CU de Strasbourg, 67404 Illkirch Cedex, France; Microarrays and Deep Sequencing Platform, Institut de Génétique et de Biologie Moléculaire et Cellulaire (IGBMC), CNRS UMR 7104, INSERM U964, UdS, BP 10142, CU de Strasbourg, 67404 Illkirch Cedex, France

**Keywords:** Histone acetyltransferase (HAT), KAT5, H3K27ac, H3K4me3, Enhancers, Super enhancers, Mouse, Pluripotency, Bivalent genes, c-Myc, Mof, NSL, MSL

## Abstract

**Background:**

Tip60 (KAT5) is the histone acetyltransferase (HAT) of the mammalian Tip60/NuA4 complex. While *Tip60* is important for early mouse development and mouse embryonic stem cell (mESC) pluripotency, the function of Tip60 as reflected in a genome-wide context is not yet well understood.

**Results:**

Gel filtration of nuclear mESCs extracts indicate incorporation of Tip60 into large molecular complexes and exclude the existence of large quantities of “free” Tip60 within the nuclei of ESCs. Thus, monitoring of Tip60 binding to the genome should reflect the behaviour of Tip60-containing complexes. The genome-wide mapping of Tip60 binding in mESCs by chromatin immunoprecipitation (ChIP) coupled with high-throughput sequencing (ChIP-seq) shows that the Tip60 complex is present at promoter regions of predominantly active genes that are bound by RNA polymerase II (Pol II) and contain the H3K4me3 histone mark. The coactivator HAT complexes, Tip60- and Mof (KAT8)-containing (NSL and MSL), show a global overlap at promoters, whereas distinct binding profiles at enhancers suggest different regulatory functions of each essential HAT complex. Interestingly, Tip60 enrichment peaks at about 200 bp downstream of the transcription start sites suggesting a function for the Tip60 complexes in addition to histone acetylation. The comparison of genome-wide binding profiles of Tip60 and c-Myc, a somatic cell reprogramming factor that binds predominantly to active genes in mESCs, demonstrate that Tip60 and c-Myc co-bind at 50–60 % of their binding sites. We also show that the Tip60 complex binds to a subset of bivalent developmental genes and defines a set of mESC-specific enhancer as well as super-enhancer regions.

**Conclusions:**

Our study suggests that the Tip60 complex functions as a global transcriptional co-activator at most active Pol II promoters, co-regulates the ESC-specific c-Myc network, important for ESC self-renewal and cell metabolism and acts at a subset of active distal regulatory elements, or super enhancers, in mESCs.

**Electronic supplementary material:**

The online version of this article (doi:10.1186/s13072-015-0039-z) contains supplementary material, which is available to authorized users.

## Background

The histone acetyl trasferase (HAT), Tip60 (Tat interactive protein 60 kDa, also called KAT5) belongs to the MYST family of HATs that play key roles in acetylation of histones and other nuclear factors and thus influence chromatin structure and transcription regulation in the eukaryotic nucleus [1]. The defining feature of the MYST family of HATs is the presence of the highly conserved MYST domain, composed of an acetyl-CoA binding motif and a zinc finger [2]. The majority of cellular Tip60 exists in a stable nuclear multiprotein complex, called the mammalian Tip60 complex, consists of at least 18 subunits and performs most transcription- and DNA damage-related Tip60 functions [3, 4]. The yeast (y) homologue of Tip60 is the yEsa1 HAT that is a subunit of the yNuA4 complex [5]. This yNuA4 HAT complex, as well as the human Tip60-containing complex, contains a large number of homologue subunits [6, 7]. In addition, the mammalian Tip60 complex seems to combine the functions of the yNuA4 HAT and the ySWR1 ATP-dependent chromatin remodelling complexes into a single complex [8]. The ATPase p400, belonging to the SWI2/SNF2 class of ATP-dependent chromatin remodelers [9], is an E1A-interacting protein essential for E1A-dependent apoptosis and cellular transformation [10]. The isolated mammalian Tip60 complexes were suggested to be heterogeneous, with a population that would contain p400 and another that would not, suggesting a dynamic assembly of the p400-containing Tip60 complex [7, 9, 11].

Tip60 complexes have three enzymatic functions: (1) a histone H2A/H4 acetlytransferase activity, (2) an ATP-dependent H2AZ.H2B dimer exchange activity and (3) a helicase activity [7, 8]. Several studies have shown that Tip60/NuA4-type complexes are involved in diverse cellular processes including transcription, cell cycle control, apoptosis, cell proliferation and DNA repair [4]. Mammalian Tip60 has been described as a transcriptional co-activator complex that is supposed to mediate the action of large variety of transcription factors, including nuclear receptors, c-Myc, STAT3, NF-kappaB, E2F1, p53 and others [4]. Importantly, a mass-spectrometry based study demonstrated that the intact Tip60-p400 (NuA4) HAT complex interacts with Myc and suggested that histone 3 and 4 acetylation patterns may be generated in part by interactions of Myc with the Tip60-p400 complex through Tip60 in mouse embryonic stem cells (mESCs) [12].

Homozygous knockout of the *Tip60* gene in mouse results in pre-implantation lethality at embryonic day 3.5 [13]. Additionally, seven subunits of the Tip60 complex, including Tip60 and p400, have been further identified in an RNAi screen to be required for mESC maintenance [14]. Moreover, siRNA down-regulation of six other components of the Tip60-complex exhibited the same phenotypic defects in alkaline phosphatase activity, embryonic body formation and teratoma formation as Tip60. This indicates that the whole Tip60 complex is necessary for mESC maintenance and normal mESC identity [14]. Interestingly, siRNA-based depletion of Tip60 and p400 in mESCs resulted in an impaired expression of developmental regulators and expression of these affected genes significantly overlapped with that regulated by Nanog in mESCs [14]. Chromatin immunoprecipitation (ChIP) linked to hybridization to promoter tiling arrays indicated that p400 localization correlates with H3K4me3 at both active and silent genes in mESCs [14], though no anti-Tip60 ChIP or ChIP-seq was carried out in this study. Surprisingly, mRNA expression analyses identified that only about 800 genes were differentially regulated in both Tip60 and p400 knock-down mESCs [14, 15]. Moreover, a recent study demonstrated that Flag-Tip60-containing complexes bind to active and developmental genes in mESCs [14, 15].

Interestingly, an additional HAT, Mof (males absent on the first or KAT8) was shown to be required for early mouse development and mESC pluripotency [16, 17]. Recently, it has been shown that Mof-associated complexes have overlapping and distinct roles in mESCs [18, 19]. We hypothesise that there is a complex interplay between different transcriptional co-factors and that both Tip60- and Mof- containing complexes have distinct role in mESCs. To better characterize the genome-wide action of the Tip60 complex, we carried out an anti-Tip60 ChIP experiment coupled to high-throughput sequencing (ChIP-seq) in mESCs. Our data demonstrate that the Tip60 complex is present at all active promoters and a subset of well-defined mESC-specific enhancer sites, suggesting that mouse Tip60 complex plays a very broad role in regulating the gene expression programmes necessary for mESC maintenance.

## Results

### The Tip60 complex acts mainly in large molecular complexes and is enriched at active promoters in mESCs

**Fig. 1:**
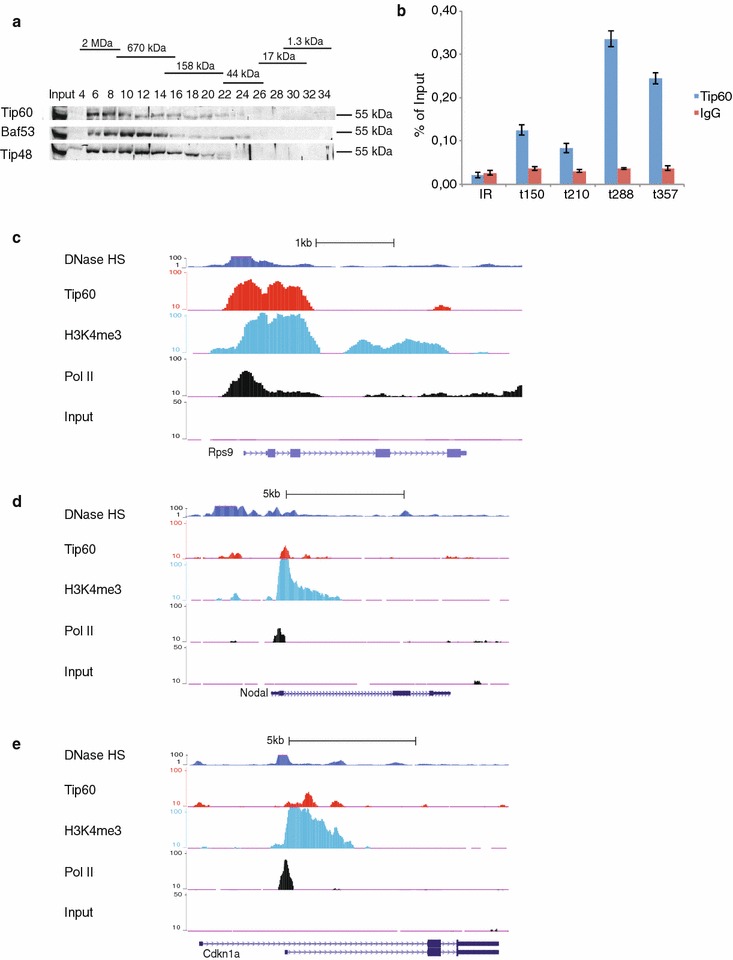
Tip60 binds to promoters as a complex in mESCs. **a** Gel filtration of mESC nuclear extracts. Every second fraction eluted from a Superose 6 column was analysed for the presence of Tip60, together with Tip49 and Baf53α by Western Blot. Native molecular weight markers eluting in the corresponding fractions are indicated on the *top* *of the panel*. **b** ChIP-qPCR validation of the ChIP-seq data in mESCs using purified anti-Tip60 antibodies [20] and a negative control IgG antibody. Primers were designed at randomly selected MACS14 peaks with different tag densities (*t*), as indicated. An intergenic region (IR) without Tip60 binding was selected as an additional negative control. The *graph* represents the results obtained in two biological replicates (with three technical qPCR replicates each). Standard deviations are indicated. **c**–**e** Tip60 binding profiles (GSE69671) together with DNAse I hypersensitive sites (DHS) (GSM1014154), H3K4me3 (GSM307618) and Pol II (GSM307623) binding are shown at three selected genes (*Rps9, Nodal and Cdkn1a*) as demonstrated by the UCSC genome browser. The input (GSM798320) serves as control

In order to investigate whether Tip60 acts mainly in large molecular complexes in mESCs, nuclear extracts were prepared and subjected to gel filtration that allows separation of macromolecules of different sizes. The analysis of the gel filtration by western blot indicated that Tip60 is present mainly in fractions eluting around 2 MDa that may correspond to endogenous Tip60 complex (about 1.3 MDa) (Fig. 1a). Moreover, in these fractions Tip60 is present together with two other Tip60 complex subunits, Tip48 (or RuvBl2) and Baf53a [7]. Note that the three subunits are also present in smaller size fractions, but less abundantly (Fig. 1a). Importantly, Tip60 is only detectable at very low levels in fractions eluting around 60 kDa, suggesting that there is very little ‘free’ Tip60 in mESCs. These results indicate that Tip60 binding profiles will mainly represent the genome-wide binding of Tip60-containing complexes.

To gain more insights into the genome-wide function of Tip60 in mESCs, we have generated high quality ChIP-seq data using previously characterized purified polyclonal anti-Tip60 antibodies that specifically recognize endogenous Tip60 [20]. Using MACS14 algorithm [21], we determined high-confidence binding sites for Tip60. The validation of several randomly selected ChIP-seq positive sites by ChIP-qPCR indicated specific Tip60 enrichments at these sites, when compared to control IgG ChIP signals and to background enrichment at an intergenic region negative for Tip60 binding (Fig. 1b).

Next, we verified our genome-wide ChIP-seq Tip60 binding data at known Tip60-regulated genes [11, 14]. We also compared Tip60 binding at these genes with available data for DNase I hypersensitive sites (DHSs), H3K4me3 and RNA polymerase II (Pol II) profiles that are markers of open chromatin and active transcription. Importantly, Tip60 is enriched at these previously described target gene promoters (Rps9, Nodal and Cdkn1 [11, 14]), together with DHSs, Pol II binding and histone H3K4me3 mark (Fig. 1c–e). These results, together with the ChIP-qPCR validation (Fig. 1b), indicate that the obtained anti-Tip60 ChIP-seq signal is specific.

**Fig. 2:**
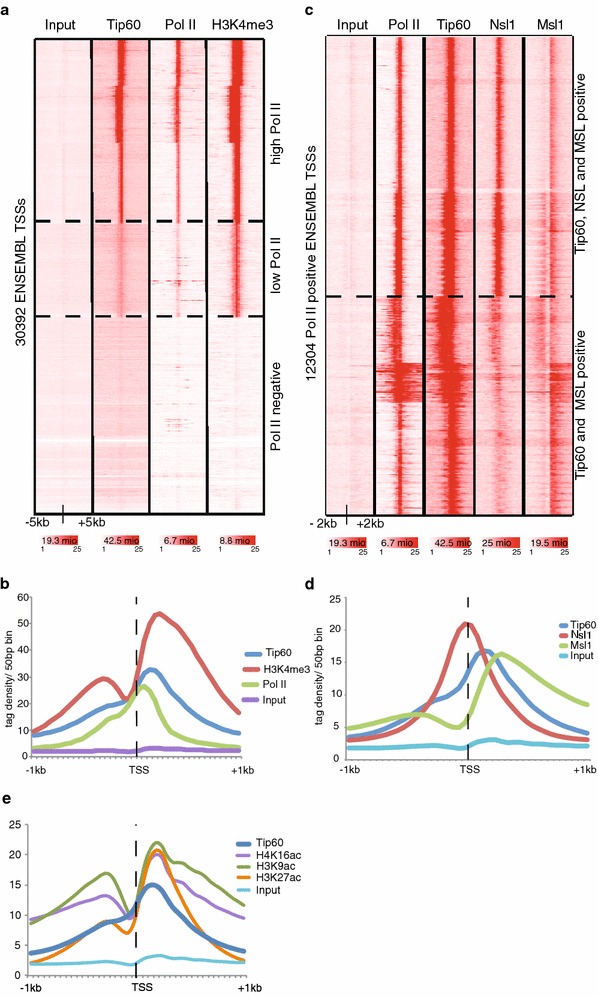
Tip60 locates to Pol II positive genes. **a** The heatmaps represents k-means clustering of Tip60, Pol II (GSM307623) and H3K4me3 (GSM307618) binding at all ENSEMBL TSSs. The Input (GSM798320) serves as control. Three main clusters are observed, as indicated. Tip60 is enriched at all Pol II and H3K4me3 positive promoters. **b** Average binding profiles of Tip60, H3K4me3 and Pol II in a region of ∓1 kb around active TSSs are depicted. Reads were normalized to Input. The input serves as control. **c** After extracting all Pol II positive ENSEMBL TSSs, *k*-means clustering was performed using Tip60 (GSE69671), Pol II (GSM307623), Nsl1 (GSM1300940) and Msl1 (GSM1300939) data sets. **d** Average binding profiles of Tip60, Nsl1 and Msl1 in a region of ∓1 kb around active TSSs are depicted. Reads were normalized to input. The input (GSM798320) serves as control. The *colour scale bars* under each data set in **a** and **c** reflect the read densities between 1 and 25 of the given dataset. The number of reads of each dataset is indicted in the *colour scale bars* in millions (mio) of reads. **e** Average binding profiles of Tip60, H4K16ac (GSM1056596), H3K9ac (GSM775313) and H3K27ac (GSM594578) in a region of ∓1 kb around active TSSs was calculated. Reads were normalized to input. To be able to better compare the datasets, the H3K27ac tag densities were divided by five. The Input (GSM798320) serves as control

To analyze Tip60 binding genome-wide around all mESC transcription start sites (TSSs), we compared the binding of Tip60 and Pol II, and the appearence of the H3K4me3 mark at all ENSEMBL TSSs [22] by k-means clustering. Interestingly, the resulting heatmap shows that Tip60 is enriched at virtually all Pol II and H3K4me3 positive promoters (11719) in mESCs (Fig. 2a).

To better characterize the genome-wide Tip60 binding around promoters, we selected all Pol II positive genes and analysed the global distribution of Tip60 around annotated TSSs (Fig. 2b). Surprisingly Tip60 peaks at about 200 bp downstream of the TSSs. Note that this Tip60 peak is slightly more downstream than the Pol II enrichment peak, which is known to be around 40–50 bps downstream of the TSSs (reviewed in [23]).

### The distinct binding profiles of Tip60, MSL and NSL complexes at TSSs suggest specific roles for these HAT complexes in transcription regulation

Genome-wide binding studies in differentiated human cells show a global co-localization of HATs and acetylated histones at transcriptional active promoters [24, 25]. The mammalian HAT Mof is the catalytic subunit of the NSL (non-specific lethal) and MSL (male-specific lethal) complex [26, 27]. We have recently analysed the genome-wide binding of two complex specific subunits Nsl1 (NSL) and Msl1 (MSL) in mESCs [18]. Since both Mof and Tip60 deletions affect mESC pluripoteny [14, 17, 28] we were further interested in the genome-wide comparison of Tip60, NSL and MSL binding at promoters. Thus, we isolated 12304 Pol II positive ENSEMBL TSSs and conducted k-means clustering. The resulting heatmap in Fig. 2c indicates Tip60, Nsl1 and Msl1 enrichment in a subset of active promoters (upper cluster), while only Tip60 and Msl1 co-localize at the second subset of promoters (lower cluster). These results suggest that the function of Tip60, NSL and MSL complexes may overlap at certain, but not all promoters.

To better dissect the function of these complexes, we compared the binding distribution of Tip60, Nsl1 and Msl1 at all Pol II positive promoters (Fig. 2d). These analyses show that Nsl1 binds directly to the TSSs, Tip60 peaks about 200 bps downstream and Msl1 even more downstream of the TSSs (in the gene bodies [18]) showing that all three HAT complexes have distinct binding profiles at promoters. These data suggest that the Tip60- and Mof-containing (NSL and MSL) complexes may not have only redundant, but also specific roles in histone acetylation, histone variant exchange and/or transcriptional regulation. Additional genome-wide comparisons between Tip60 binding and available acetylated histone H3 and H4 profiles (H3K9ac, H3K27ac and H4K16ac) show that Tip60 overlaps with these marks, but peaks slightly upstream of the analysed acetylated H3 and H4 marks (Fig. 2e).

### The large majority of Tip60 binding sites overlap with that of c-Myc

**Fig. 3:**
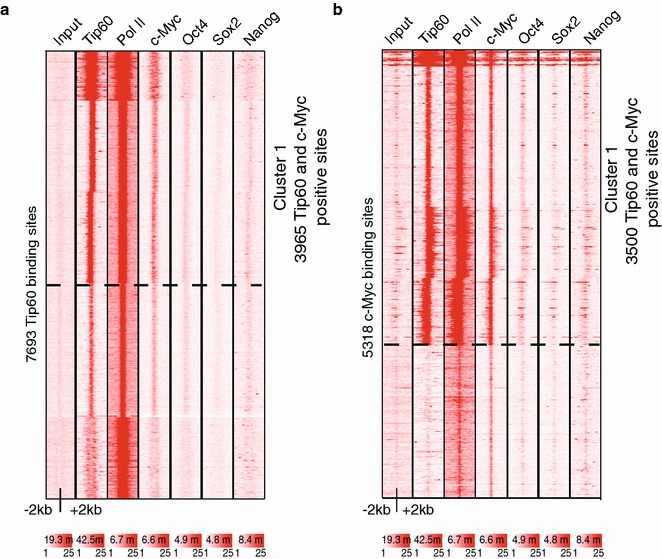
Tip60 and c-Myc binding overlap. **a**–**b** Heatmap representing *k*-means clustering results of normalized Tip60 (GSE69671), Pol II (GSM307623), c-Myc (GSM288356), Oct4 (GSM288347), Sox2 (GSM288346) and Nanog (GSM288345) density profiles against all 7693 MACS14 Tip60 peaks (**a**), or against all 5318 MACS14 c-Myc peaks (**b**). Two main clusters are observed. Cluster 1 shows Tip60 and c-Myc overlap in *both panels*. The *colour scale bars* under each data set in **a** and **b** reflect the read densities between 1 and 25 of the given dataset. The *number of reads* of each data set is indicted in the *colour scale bars* in millions (m) of reads

Tip60 is known to interact with and regulate various transcription factors as a transcriptional co-factor [4]. c-Myc is a transcription factor of the basic helix-loop-helix leucine zipper (bHLH-LZ) family, which dimerizes with another bHLH-LZ protein, Max [29]. Importantly, the oncoprotein c-Myc recruits Tip60 [20] and is regulated by the catalytic activity of Tip60 [30]. c-Myc is a somatic cell reprogramming factor (together with Oct4, Sox2, Klf4, Nanog and others) and a member of the so-called Myc regulatory module (together with n-Myc, Rex1, Zfx and E2f1) that is known to be involved in mESC self-renewal and cell metabolism [31–33]. Protein–protein interaction networks revealed that Tip60 complex interacts with c-Myc in mESCs [12]. Moreover, c-Myc binds predominantly active genes in mESCs [34] and c-Myc is known to recruit Tip60 to target promoters [35]. To better understand the genome-wide interactions between Tip60 and c-Myc at the chromatin, we analysed the overlap between Tip60 and c-Myc high-confidence binding sites, which were identified by MACS14 peak calling algorithm [21]. When either all high-confidence (7693) Tip60 peaks are compared to published c-Myc sites (Fig. 3a), or when all 5318 c-Myc peaks are compared to Tip60 sites (Fig. 3b) using k-means clustering, about 50–65 % of all Tip60 binding sites are co-bound by c-Myc and vice versa. Importantly, other mESC pluripotency factors, such as Oct4, Sox2 and Nanog, are not enriched at the c-Myc and Tip60 co-bound sites (Fig. 3b), which is in agreement with the finding that the Myc-cluster appears to function independently from the core pluripotency network [32, 34]. These results suggest that the Tip60 complex is co-bound at about 50–65 % of Myc/Max sites in mESCs and that it is directly involved in regulating the c-Myc-dependent transcriptional network.

### Tip60 locates to transcriptional active genes in mESCs

**Fig. 4:**
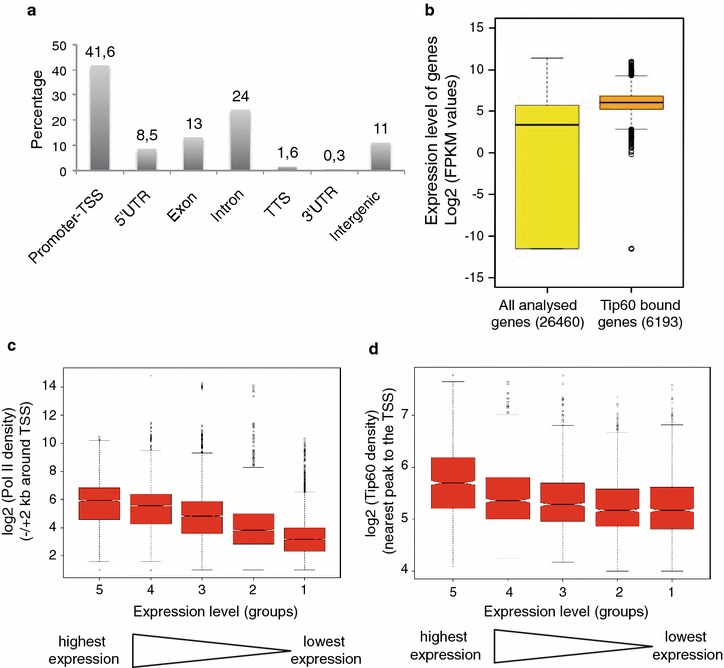
Tip60 is a positive transcriptional co-activator. **a** All the identified Tip60 peaks (100 %) were annotated to different genomic elements: Promoter-TSS, UTRs (untranslated regions), exons, introns, TSS and intergenic regions and are represented in the % of their presence at these elements. **b** *Boxplots* showing the log2 of RNA fragments per kilobase per million mapped fragments (FPKM) expression values from ESCs (GSE34473) of all analysed genes and Tip60 bound genes. **c**–**d** RNA expression values are ranked into five groups with equal number of genes from highest to lowest RNA expression level as indicated. *Boxplots* represent the Pol II densities (**c**) or the Tip60 peak density (**d**) around the TSSs. Only density values higher than zero were taken. The median is significantly different between groups, if the notches do not overlap

To further characterize Tip60 function, we categorized the binding of Tip60 to different genomic regions. When the MACS identified 7693 Tip60 binding peaks were annotated to promoter-TSS, 5′- or 3′-untranslated regions (UTRs), exons, introns and intergenic regions, about 42 % of all high-confidence Tip60 binding peaks were found at promoter-TSS regions (Fig. 4a). Interestingly, about 35 % of the binding sites were localized at either intronic (24 %) or intergenic regions (11 %), suggesting that Tip60 may also play a role in regulating enhancer activity (see below).

Next, we were interested in expression levels of Tip60 enriched genes and how Tip60 binding correlates with gene expression. To isolate Tip60 bound ENSEMBL genes, each Tip60 peak was annotated to its closest gene in a region of 1 kb up- or downstream of TSSs. Thus, we defined 6193 genes bound by the Tip60 complex. Importantly, the average expression level of all Tip60 positive genes is significantly higher compared to that of all ENSEMBL genes (Fig. 4b), suggesting that Tip60 complexes bind predominantly to active genes. As it is known that the Pol II binding strength at promoters reflects the gene expression levels [36] (Fig. 4c), we wanted to analyse whether this would be the case for Tip60 complexes. Similarly to Pol II, the Tip60 peak tag density positively correlates with the gene expression level of bound genes (Fig. 4d). Altogether our data suggest that Tip60 locates to Pol II positive and transcriptional active genes.

### Tip60 binds also to bivalent genes and active enhancer elements

**Fig. 5:**
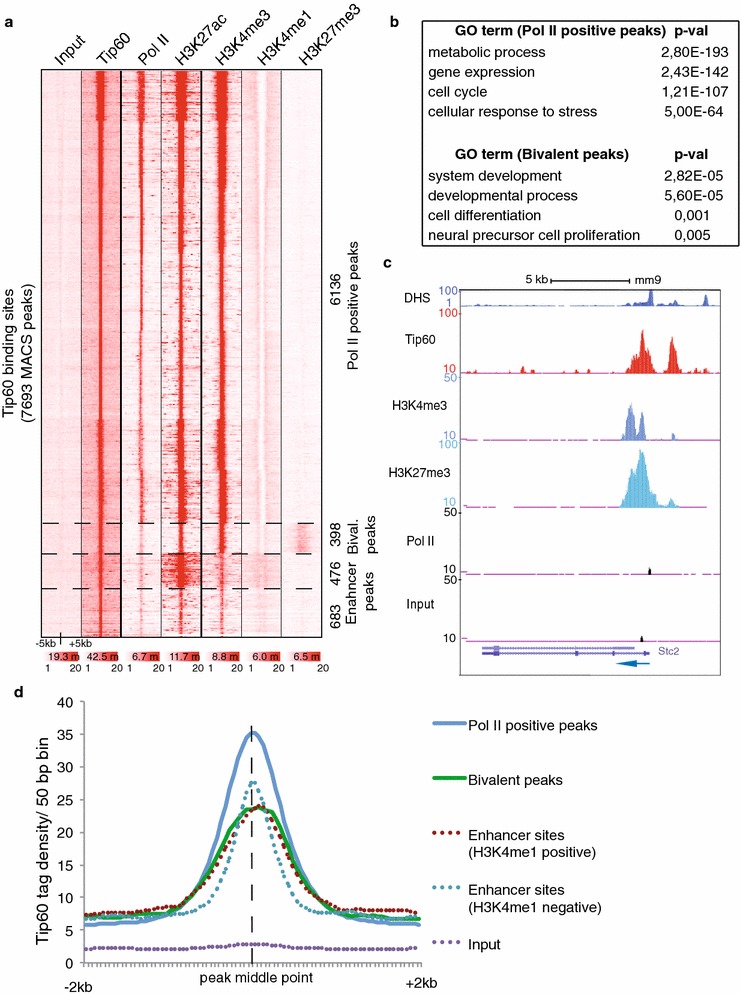
Tip60 binding defines active gene sets and enhancer regions. **a** Heatmap showing k-means clustering of Tip60 (GSE69671), Pol II (GSM307623), H3K27ac (GSM594578), H3K4me3 (GSM307618), H3K4me1 (GSM594577) and H3K27me3 (GSM307619) using 7693 high-confidence Tip60 binding sites as reference coordinates. Densities are represented in region of ∓5 kb around Tip60 binding sites. Four clusters are defined as indicted by the *dotted lines*. The two “enhancer” peaks are divided into H3K4me1 positive and H3K4me1 negative peaks. The *colour scale bars* under each data set in A reflect the read densities between 1 and 20 of the given dataset. The number of reads of each data set is indicted in the *colour scale bars* in millions (m) of reads. **b** GO term analyses of Pol II positive or bivalent peaks after gene annotation using Manteia [62]. **c** UCSC genome browser profiles of DHS, Tip60 binding, presence of H3K4me3 and H3K27me3, Pol II binding and input (negative control) at a randomly chosen bivalent gene, Stc2, are shown. An *arrow labels* the gene orientation. **d** Density profiles of Tip60 binding at the different clusters as defined in *panel* **a**

To further address the global distribution and function of the Tip60 complex in mESCs, we compared high-confidence Tip60 binding sites with marks, which are either associated with active transcription at promoters (Pol II and H3K4me3) or enriched at enhancer sites (H3K4me1 and H3K27ac) [37, 38]. We also analysed H3K27me3, which establishes, together with H3K4me3, a bivalent chromatin state in mESCs at developmental genes [39]. After conducting k-means clustering using seqMINER against all identified Tip60 peaks [22], a large colocalization with Pol II, H3K27ac and H3K4me3 enriched sites was found (6136 peaks in Fig. 5a). Our GO term analyses of Tip60 enrichment at Pol II positive genes show that these Tip60 positive genes are involved not only in biological functions such as ‘metabolic processes’ and ‘gene expression’, but also in ‘cell cycle and ‘cellular response to stress’ (Fig. 5b, upper panel). Importantly, some of these GO categories, i.e. metabolic processes’, are identical than the ones defined for c-Myc [32], in agreement with our finding that at 50–65 % Tip60- and c-Myc-bound loci these factors cooperate in gene regulation.

The heatmap in Fig. 5a further indicates that Tip60 binds to about 400 sites, which are positive for H3K4me3 and H3K27me3 and are thus defined as bivalent sites (Fig. 5a). Tip60 binding at a representative bivalent gene is further illustrated in Fig. 5c. GO term analyses of all Tip60-bound bivalent genes resulted in a significant enrichment of GO terms with developmental functions (see Fig. 5b lower panel). In agreement with observations that bivalent genes are very weakly transcribed (reviewed in [40]), Tip60 tag density at bivalent sites (green line) was lower than Tip60 enrichment observed at 'Pol II positive peaks' (blue line) (Fig. 5d). Therefore, our analyses show that Tip60 complexes locate also to bivalent or developmental genes, as suggested previously [14, 15].

Interestingly, two of the last Tip60 positive clusters within the heatmap (Fig. 5a) show low Pol II enrichment levels. One of these Tip60 positive clusters, comprising 476 sites, contains high levels of H3K4me1 and H3K27ac marks, suggesting that these sites correspond to active enhancers [37, 38]. The comparison of H3K4me1/K3K27ac positive Tip60 peak distances to TSSs of annotated genes revealed that these peaks are located at distal regulatory regions, such as active enhancers (Additional file 1: Figure S1a, b). These findings are further illustrated at a known enhancer region, (see UCSC genome browser tracks at Additional file 1: Figure S1c). The second ‘very low Pol II’ cluster may represent less active enhancers and other not well-characterized genomic regions. Nevertheless, clustering of Tip60 with the above-described well-known chromatin marks and Pol II allowed us to suggest that Tip60-containing complexes act mainly at active Pol II promoters, at bivalent genes and at active enhancers.

### Tip60 defines a subset of mESC-specific enhancer

**Fig. 6:**
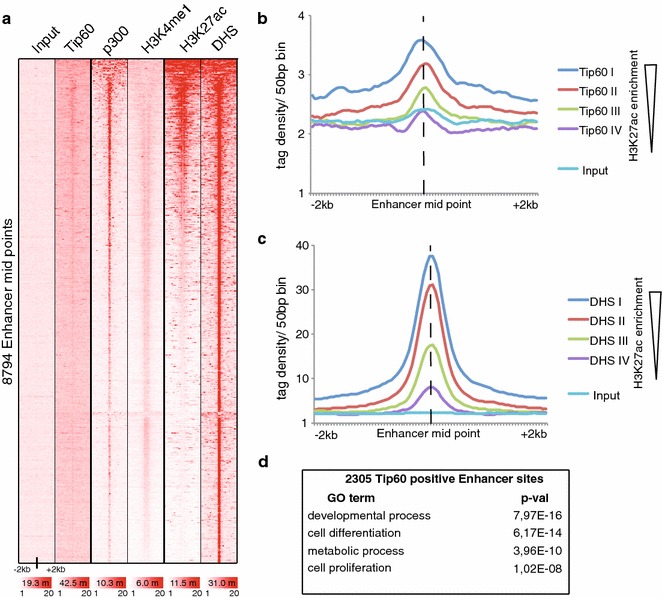
Tip60 correlates with p300, H3K3me1 and DHS presence and with a subset of H3K27ac signals at ESC-specific enhancer sites. **a** Enhancers [41] were sorted for H3K27ac (GSM594578) signal intensities. Signals of Tip60 (GSE69671), p300 (GSM723018), H3K27ac (GSM594578), H3K4me1 (GSM594577), H3K27me3 (GSM307619) and DHS (GSM1014154) were calculated around enhancer mid points. The *colour scale bars* under each data set in **a** reflects the read densities between 1 and 20 of the given dataset. The number of reads of each data set is indicted in the *colour scale bars* in millions (m) of reads. **b**, **c** Enhancers were divided into four categories having equal number of genes based on their H3K27ac signal intensity from high to low (I–IV) as indicated on the *right of the panels*. *Graphs* show Tip60 enrichment (**b**) or DHS signals (**c**) around enhancer mid points of the different categories. (**d**) GO term category analysis by Manteia [62] of the closest genes to the Tip60 positive enhancers [41] identified by *k*-means clustering

As our above analyses suggested that Tip60 complexes could bind to enhancers; we wanted to examine the total enrichment of Tip60 at all known enhancer sites. To this end, we have taken annotated enhancer sites from mESCs [41]. Since active enhancers often have high H3K27ac levels [37], enhancers were sorted for H3K27ac signal intensity and analysed for enrichment of Tip60, p300, H3K4me1 and DHS. Interestingly, on these enhancer sites Tip60 binding showed a co-occurence with p300, H3K4me1 and DHSs and a partial overlap with a subset of sites having high H3K27ac signals (Fig. 6a). Next, we divided these H3K27ac labelled enhancers into four equal clusters (from high to low H3K27ac signals) and average tag densities for Tip60, as well as for DHS profiles, around enhancer mid points of each cluster were calculated (Fig. 6b, c). Importantly, Tip60 and DHS enrichment have a positive correlation with each other and with that of H3K27ac. To define the number of enhancers that are positive for Tip60 and all the other enhancer defining marks (H3K4me1, H3K27ac and DHS), k-means clustering was performed to analyse the presence of Tip60, p300, H3K4me1, H3K27ac and DHS at these defined enhancer sites [41]. The heatmap analysis in Figure S2A clearly shows that 2305 enhancers defined in cluster 1 are positive for Tip60 and all the other enhancer chromatin marks (p300, H3K27ac and H3K4me1). In contrast, the 6489 enhancers in cluster 2 show almost no Tip60 enrichment (Additional file 2: Figure S2b, c). Moreover, GO terms of genes in the vicinity of the 2305 Tip60 bound enhancers are associated with developmental processes (Fig. 6d). Thus, it seems that Tip60 is recruited to about 26 % of all mESC-specific enhancers.

**Fig. 7:**
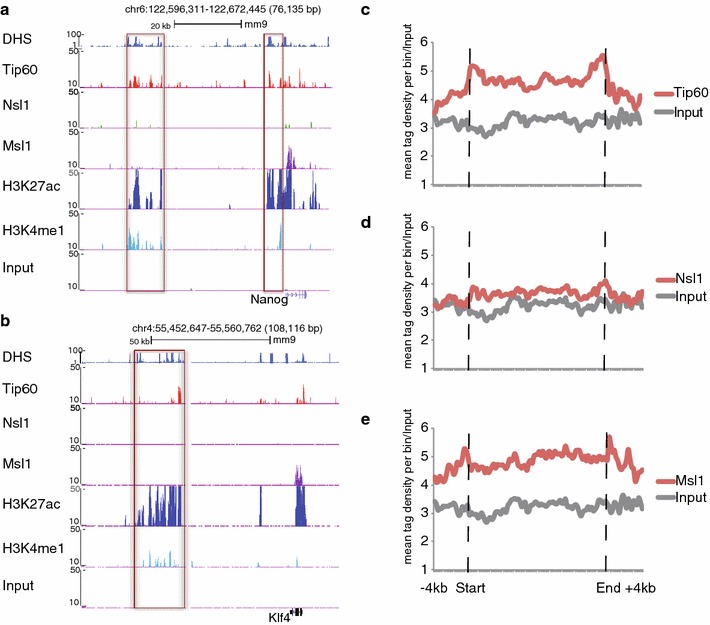
Tip60 locates to super enhancer regions. **a** Tip60 (GSE69671) binding profiles together with DHS (GSM1014154), H3K4me1 (GSM594577), H3K27ac (GSM594578), Nsl1 (GSM1300940) and Msl1 (GSM1300939) are represented using the UCSC genome browser at the well-defined super enhancer regions (*boxed in red*) of the *Nanog* and *Klf4* locus. The Input (GSM798320) serves as negative control. Data were uploaded as wig files. **b**–**d** The 231 super enhancers [41] were divided into 80 bins and Tip60 (**b**), Nsl1 (**c**) and Msl1 (**d**) enrichment was calculated per bin between their start and end positions as well as up to 4 kb up- and downstream. Signals were normalized to the input (GSM798320)

As two of the so-called ‘super enhancer’ regions of the Nanog gene and the enhancer of the Klf4 gene are bound by Tip60, but not by Nsl1 and Msl1 (Fig. 7a, b), we further characterized Tip60 binding at enhancer regions in detail by analysing Tip60 presence at the previously defined 231 ‘super enhancer’ regions [41]. We observed Tip60 enrichment (compared to the Input control) between the start and end positions of these 231 ‘super-enhancers’ (Fig. 7c; Additional file 2: Figure S2d). However, global analyses of Mof-containing complexes at these ‘super-enhancer’ regions displayed Msl1, but no or very weak Nsl1 enrichment (Fig. 7d, e). Altogether our data show that Tip60 is recruited to a subset of active enhancers out of which certain have been defined as super enhancers in mESCs.

## Discussion

In this study, we analysed the genome-wide binding of Tip60-containing complexes to understand their role in transcription regulation in mESCs. Our gel filtration analyses show that Tip60 incorporates into large molecular complexes in mESCs, as previously described in other systems, and that heterogeneous populations of Tip60-containing complexes exist, which might dynamically change their association with p400 and/or other subunits. However, our observations that there is very little free Tip60 within the nuclei of mESCs indicates that the ChIP binding profiles obtained with anti-Tip60 antibodies represent mostly the behaviour of the corresponding endogenous Tip60 complexes.

Our study demonstrates that the majority of genome-wide Tip60 binding occurs at promoter regions, where it co-localizes with Pol II, H3K4me3 and DHS sites. Importantly, Tip60 bound genes are expressed and the Tip60 enrichment positively correlates with gene expression levels. Interestingly, using an unbiased clustering method of all mapped Tip60 reads against ENSEMBL TSS, and we observed that all transcriptionally active genes (Pol II positive) show Tip60 enrichment. Genome-wide binding and knock-down studies of Tip60-p400, NSL and MSL HAT complexes reported the binding of these complexes at Pol II positive genes and in mESCs [18, 19]. When directly comparing binding profiles of Tip60- and Mof-containing complexes around Pol II positive promoter regions, we have found a global overlap at Pol II positive mRNA coding genes, whereas each of the HAT complexes has a distinct binding profile around the TSS. Altogether, this suggests that (1) Tip60- and Mof-containing HAT complexes globally regulate gene expression though their presence at promoters (NSL and Tip60) and further downstream of promoters (i.e. MSL) and (2) that there might be a different function of each HAT complex in histone acetylation, histone exchange and transcriptional regulation.

The frequent overlap of Tip60 and c-Myc binding strongly indicates a role of Tip60 complexes within the Myc-centred regulatory pluripotency network in mESC [31, 33]. Moreover, Tip60 complexes bind and possibly regulate a subset of active enhancers and super enhancers, revealing an additional layer of regulation by which Tip60 complexes influence mESC maintenance.

Tip60 complexes preferentially acetylate histone H4 [8]. Acetylation of several histone H3 and H4 lysines was described to be up-regulated at the majority of Myc-target promoters [42]. Several studies in *Drosophila,* mESCs and human cells have revealed that c-Myc targets at least 10–15 % of all cellular promoters [35]. Nevertheless, it has been suggested that although Myc can bind to a large number of genes, it is critical for the regulation of a subset of those genes depending on (1) protein–protein interactions between Myc/Max dimers, such as chromatin-bound protein complexes [43] and (2) the cellular or physiological context [44]. Our finding that at least half of the c-Myc binding sites are co-bound by the Tip60 complex suggests that Myc/Max and the Tip60 complex may cooperate to stabilize each other’s binding. Moreover, at these co-bound sites the Tip60 complex is thought to be involved in acetylation of promoter-associated nucleosomes that can participate in transcriptional activation [35].

Interestingly, the genome-wide Tip60 complex binding profile peaks at regions that are about 200 bps downstream of TSSs (Fig. 2b). It is, therefore, conceivable that this downstream binding may reflect an additional function of the Tip60 complex that may play a role in the early steps of transcription elongation processes, as suggested also in *Drosophila* [45]. Interestingly, c-Myc is believed to control the release of Pol II from promoter proximal transcriptional pause [44]. Thus, the Tip60 complexes bound 200 bps downstream of the TSS at many active genes may cooperate with c-Myc to co-regulate Pol II pause release and consequently, transcriptional elongation in mESCs.

Moreover, our results indicate that Tip60 complexes may be involved in gene expression regulation by occupying a set of previously identified ESC-specific enhancers [41]. Tip60 enrichment correlates with H3K27ac levels and chromatin accessibility (DHS sites). Genes associated with enhancers with increased Tip60 binding (determined by *k*-means clustering) are predicted to be involved in regulation of development, metabolism and proliferation of mESCs. Based on high Mediator occupancy, some of these enhancers can be clustered to 231 super-enhancer regions [41], which likely control important genes for stem cell maintenance (i.e. the Nanog or Klf4 gene). Importantly, these super-enhancers show Tip60 and Msl1 occupancy, but have only very little Nsl1 binding. The fact that Tip60 possibly regulates a subset of active enhancers and super enhancers reveals an additional layer of regulation by which Tip60 complexes influence mESC maintenance. Note that previously we have described enhancers, which were bound by the ATAC HAT complex but not by p300 in differentiated human cells [46]. Thus, it is possible that different enhancers are bound by a given combination of HAT complexes to regulate their activity.

Human Tip60 is known to play a wide role in transcriptional regulation [47]. The identified Tip60 bound genes belong to gene sets with housekeeping and developmental functions. Other studies further suggested that the Tip60 complex might have a repressive function at low expressed bivalent/developmental genes [14, 15]. Since the Tip60 complex has different catalytic activities by acetylating histones or exchanging H2A.Z, which further depend on each other, it will be challenging to understand the molecular mechanism by which the Tip60 complexes regulate transcription at these low-expressed genes in mESCs. Nevertheless, it is tempting to speculate that the Tip60 complex is recruited to these genes to poise these genes that will be important for their rapid activation during cellular differentiation.

Our genome-wide binding analyses suggest that the Tip60 complex has a very broad role in regulating transcription in mESCs, which is in agreement with the observation that Tip60 and several other Tip60-complex subunits are required for mESC maintenance [14]. In contrast, Tip60 knock-down studies identified less than 900 differentially expressed genes when analysing steady-state mRNAs [14, 15]. The potential reason for this apparent contradiction may be that the previous Tip60 knock-down studies analysed steady-state mRNAs instead of newly synthetized transcripts. Recently, it has been demonstrated that the sole analysis of polyA + mRNAs may not give any information on the direct transcriptional output of a given transcription factor/co-activator complex, as cells can buffer global transcription changes by adjusting mRNA decay in parallel ([48–50] and refs therein). Thus, novel newly synthetized RNA detection methods are needed to address global effects of Tip60 on newly synthetized transcripts, such as in vivo RNA labelling with 4-thiouridine (4sU) [49] or analyses of profiles of transcriptional active Pol II (i.e. NET-seq; [51]) under ∓ Tip60 conditions. Furthermore, the understanding of how the different activities (acetylation, histone variant exchange and/or nucleosome remodelling activity) of the Tip60 complexes influence transcription at promoters and enhancers in mESCs will be an equally important task.

## Conclusions

Overall, we establish that Tip60-complexes are present at promoter regions of active RNA polymerase II genes and that half of Tip60 binding sites overlap with binding of the somatic cell reprograming factor, c-Myc, known to regulate an ESC specific transcriptional module. Importantly, Tip60, NSL and MSL coactivator HAT complexes have a genome-wide overlap at many active genes, but their specific functions might be reflected in their distinct binding profiles around the TSSs. Moreover, Tip60 complexes define a subset of bivalent developmental genes and a subset of ESC-specific enhancers. Thus, our study suggests that the Tip60 complex is important for mESC pluripotency by acting as a global transcriptional co-factor at active genes and distal regulatory elements.

## Methods

### Cell culture

The ES cell line, E14 was derived from 129P2/OlaHsd strain blastocysts [52] and cells were cultivated on 0.1 % gelatine (Sigma, France) and CD1 feeder cells (37 °C, 5 % CO_2_) in DMEM (4,5 g/L glucose/w-Glutamax) (Invitrogen, France), 15 % foetal calf serum (PAA), 5uM LIF, 50 mM ß-mercaptoethanol (Invitrogen, France), penicillin (10,000 U/ml) and streptomycin (10 mg/ml) (Invitrogen, France), 200 mM l-glutamine (Invitrogen, France) and 1× non-essential amino acids (GIBCO, France). To work under feeder-free conditions cells were treated with 1 mg/ml Collagenase and 2 mg/mL Dispase (GIBCO, France) and cultivated for one passage without feeder cells on 0.1 % gelatine plates. Experiments were conducted with E14.wt cells at passages 26–29.

### Chromatin immunoprecipitation coupled to Solexa sequencing (ChIP-seq)

ChIP was carried out as described in [18]. For 500 μg of chromatin 6 μg of the well-characterized rabbit anti-Tip60 antibody mixture (RLPV, CLGT and CLHF purified polyclonal sera) was used [20]. 8 ng of precipitated DNA from Tip60 ChIP was used for Solexa sequencing. Rabbit anti-IgG antibody (ab37415) was purchased from Abcam. To create a genomic library the instructions of NEBNext protocol (E6240, Biolabs) was followed. The library was validated with the Agilent Bioanalyzer. Single reads run sequencing was done with Illumina HiSeq 2000. Image analysis and base calling were conducted with the Illumina pipeline (1.8.2). The July 2007 Mus musculus genome assembly (NCBI37/mm9) from NCBI was used for the sequence alignment by the software Bowtie (0.12.7) [53]. The analysis was conducted with unique reads. Read density (wig) files were created out of bed files by extending reads to 200 bp length and creating 25 bp bins. To detect ChIP-seq peaks the MACS14 peak-calling algorithm was applied using default parameters [21]. The Tip60 ChIP-seq data were deposited in the Gene Expression Omnibus (GEO) databank under the accession number GSE69671.

For further analyses the following ChIP-seq files were included from Gene Expression Omnibus(http://www.ncbi.nlm.nih.gov/geo/): Input (GSM798320) [54], RNA polymerase II (GSM307623), H3K4me3 (GSM307618), c-Myc (GSM288356), Oct4 (GSM288347), Sox2 (GSM288346) and Nanog (GSM288345) [31], H3K4me1 (GSM594577), H3K27ac (GSM594578) [37], H3K9ac (GSM775313) [54], H4K16ac (GSM1056596) [55], p300 (GSM723018) [56], H3K27me3 (GSM307619) [57], Nsl1 (GSM1300940) and Msl1 (GSM1300939) [18]. Tip60 data are deposited in GEO under the following accession number: GSE69671. Fastq files were generated from SRA lite format and aligned to the NCBI37/mm9 assembly using Bowtie (0.12.7) [53]. DNAse I hypersensitive sites were taken from Encode/UW (GSM1014154). Detailed information summarizing all the used ChIP-seq files is presented in Additional file 3: Table S1.

### ChIP-qPCR

The Tip60 ChIP-seq was validated by ChIP coupled to quantitative PCR (ChIP-qPCR). Identified MACS14 Tip60 peaks were randomly taken based on different tag densitites (t). SYBR Green (Roche) was used according to the manufacturers protocol. Following primers were designed:

**Table:** 

t150_fw	TGATCGGCGCAGAGACAAGA
t150_rv	ACAAAAGGCCCCTCCTTGCT
t210_fw	TCGCTTTGCAGCAGTGAGATG
t210_rv	TGGCCTCGGACCTTTCAATC
t288_fw	CGGCTTCGGGGTTTTCTTTT
t288_rv	TTATCCCATTCCGGGAGACG
t357_fw	ACCAGGTCCTCGGCGATAGTTT
t357_rv	CTTTCCTCGCGGATCGAAGA
intergenic_fw	TGATGCAACACATGGACATTTCTG
Intergenic_rv	TTCAGGGGTTGGGACAAAGTG

### Gel filtration

The gel filtration experiment using a Superose 6 column was described in [18]. Input nuclear extract and every second fraction eluting from the column were tested by western blot assays using the Tip60 antibody mixture [20] at dilution 1:2000, the anti-Tip48 or anti-Baf53α antibodies [58] at dilution 1:500.

### Bioinformatics analyses of Tip60 ChIP-seq in mESCs

Density profile calculation around TSSs as well as *K*-means linear clustering was conducted with seqMINER [22]. *K*-means clustering was performed with normalized read densities, while resulting heatmaps show total number of reads. Obtained MACS14 peaks were annotated using the software HOMER [59] based on the ENSEMBL 67 database (mm9).

To determine the Tip60 or Pol II enrichment at genes, the peak tag density of the nearest peak to the TSS (in a region of +2 kb) was taken and correlated with gene expression levels. We considered a total of 26,460 ENSEMBL TSSs based on the ENSEMBL 67 database (mm9). For this, raw RNA-seq data of mESCs from Gene Expression Omnibus (GSE34473) were processed using the software tools TopHat [60] and HTSeq with default parameters. FPKM (fragments per kilobase of exon per million fragments mapped) values were calculated with Cufflinks [61]. FPKM values were correlated with Tip60 and Pol II enrichment and taken to analyse average expression levels of Tip60 bound genes.

Gene ontology (GO) term analyses of Pol II positive or bivalent peaks as well as enhancer sites were conducted with Manteia [62]. Peaks were annotated to nearest promoters [59] prior GO term analysis.

ES-specific enhancers and super enhancer regions were taken from [41]. ES-specific enhancers were ranked according to H3K27ac signal intensities. Enhancer-midpoints were calculated for further analysis. The 231 total super enhancers were divided into 80 bins from start to end positions and the mean Tip60 and Input read densities were calculated for each bin. Moreover, read densities of regions 4 kb down-or upstream of super enhancer start or end positions were determined for each 50 bp bin. Total Number of reads is normalized to the Input.

## Additional files

10.1186/s13072-015-0039-z Tip60 defines bivalent genes and enhancer sites. (**A** and **B**) Mapping of Tip60/Pol II positive (**A**) or Tip60/H3K27ac/H3K4me1 positive peaks to TSSs and more and more distal genomic regions. X-axis indicates distances of peaks to TSSs. (**C**) Tip60 enrichment at putative enhancer sites, which are H3K4me1 (GSM594577) and H3K27ac (GSM594578) positive at the UCSC genome browser. The Input (GSM798320) is the negative control.
10.1186/s13072-015-0039-z Tip60 is enriched at ESC-specific enhancer sites. (**A**) Heatmap showing k-means clustering of Tip60, p300 (GSM723018), H3K27ac (GSM594578) and H3K4me1 (GSM594577) using enhancer mid points [41] as reference coordinates. Two main clusters were defined: one with significant Tip60 binding (cluster 1), and a second where Tip60 cannot be detected (cluster 2). Densities are represented in region of ∓ 2 kb around enhancer mid points. The representation is as in Fig. 1a. (**B** and **C**) Tip60 enrichment around enhancer mid points of cluster 1 (**B**) and cluster 2 (**C**). Clusters were defined in panel A.
10.1186/s13072-015-0039-z. Summary of all ChIP-seq datasets used representing the number of unique reads and GEO accession numbers.

## Abbreviations

Ac: acetyl
bHLH-LZ: basic helix-loop-helix leucine zipper
bp: base pairs
ChIP: chromatin immunoprecipitation
ChIP-seq: chromatin immunoprecipitation coupled to sequencing
DHS: DNase Hypersensitive Site
GEO: gene expression omnibus
H: histone
HAT: histone acetyltransferase
KAT: lysine acetyltransferase
K: lysine
kb: kilobase
kD: kilo Dalton
m: mouse
me: Methyl
mD: mega Dalton
mESCs: mouse embryonic stem cells
Mof: male absent on the first
MSL: male-specific lethal
mRNA: messenger RNA
NSL: non-specific lethal
Pol II: RNA polymerase II
qPCR: quantitative polymerase chain reaction
shRNA: small-hairin RNA
siRNA: small interfering RNA
Tip60: tat-interacting protein 60 kDa
TSS: transcription start site
UTR: untranslated-region
y: yeast
WB: western Blot
